# Retraction: Exosomal CD44 transmits lymph node metastatic capacity between gastric cancer cells via YAP-CPT1A-mediated FAO reprogramming

**DOI:** 10.3389/fonc.2026.1961011

**Published:** 2026-08-24

**Authors:** Frontiers Editorial Office

The journal retracts the 10 March 2022 article cited above.

Following publication, concerns were raised regarding the integrity of the images in the published figures. Image duplication concerns were identified in Figure 4E and 5B.

The authors failed to provide a satisfactory explanation during the investigation, which was conducted in accordance with Frontiers’ policies. As a result, the data and conclusions of the article have been deemed unreliable, and the article has been retracted.

This retraction was approved by the Chief Editors of Frontiers in Oncology and the Chief Executive Editor of Frontiers. The authors agree to this retraction.

Frontiers would like to thank Kevin Patrick for contacting the journal regarding the published article.

